# Paediatric clinical research in Europe: an insight on experts’ needs and perspectives

**DOI:** 10.1016/j.conctc.2021.100735

**Published:** 2021-02-10

**Authors:** Lucia Ruggieri, Adriana Ceci, Franco Bartoloni, Valèry Elie, Mariagrazia Felisi, Evelyne Jacqz-Aigrain, Mariangela Lupo, Salma Malik, Cristina Manfredi, Giorgio Reggiardo, Jacques Demotes, Donato Bonifazi

**Affiliations:** a Fondazione per la Ricerca Farmacologica Gianni Benzi Onlus, Via Abate Eustasio 30, 70100, Valenzano, BA, Italy; b Consorzio per Valutazioni Biologiche e Farmacologiche, Via Nicolò Putignani 178, 70122, Bari, Italy; c Assistance Publique Hôpitaux de Paris, Clinical Investigation Center, Hôpital Universitaire Robert Debré, 48 Boulevard Serrurier, 75019, Paris, France; d TEDDY - European Network of Excellence for Paediatric Research, Via Luigi Porta, 14 27100, Pavia, Italy; e The European Clinical Research Infrastructure Network (ECRIN), 5-7 Rue Watt, 75013, Paris, France

**Keywords:** Paediatric, Research infrastructure, Clinical trials network, Research need, Survey

## Abstract

PedCRIN is a Horizon 2020 project aimed to develop a paediatric component of ECRIN (European Clinical Research Infrastructure Network) including tools supporting the conduct of neonatal and paediatric trials.

A structured, cross-sectional, closed-ended questionnaire was electronically administered from April to May 2017 to stakeholders involved in paediatric clinical research to capture their needs to receive infrastructural support to cover specific research gaps. The questionnaire included 6 headings and 29 subheadings. Each item was evaluated using a Likert-scale.

147 questionnaires were returned (response rate of 24.6%). The application of innovative study design and the preparation of protocols for paediatric interventional clinical trials had the highest frequency of high need for support (123 and 117 respondents, respectively). Similarly, the identification and applications to relevant calls for funding was acknowledged as an area in which support is needed (123 respondents declaring high need).

In 14 out of 29 activities, need for support was significantly higher in the respondents not being part of a Paediatric Research Network or Consortium (especially for regulatory expertise, pharmacovigilance and GCP training).

Conclusions: These results document that the achievement of PedCRIN objectives would greatly advantage the paediatric research community.

## 1 Introduction

An appropriate developmental process guarantees that paediatric medicines use is supported by high quality and ethical research, generating valuable data on medicines efficacy and safety in all the concerned age subsets. The generation of evidence through clinical trials is impaired by several obstacles: methodological failings contributing to the wasting of about 85% of biomedical clinical research [1], difficulties in recruitment and retention of participants, financial costs, insufficiencies in the clinical research workforce, regulatory and administrative barriers, distance between clinical research and medical care, ethical issues [2,3,4]. General challenges and difficulties in paediatric research are reported in literature [5,6] and it has been estimated that 19% of paediatric trials registered in clinicaltrials.gov from 2008 to 2010 discontinued because of several reasons (patient accrual, conduct problems, company decision), for a total of 8369 children enrolled in unfinished trials [7]. Recruitment difficulties are the most common challenge in paediatric clinical trials: they deferred the completion of 167/365 Paediatric Investigation Plans up to 2015, with similar trends in the following years [8].

However, a systematic description of these gaps is still lacking.

Multinational clinical trials offer advantages in terms of recruitment capacity, but they are more difficult because of administrative requirements and procedures.

Several measures have been adopted to improve the conduct of paediatric clinical trials [7], ranging from age-appropriate procedures for clinical trials, recommendations for the use of suitable medicine forms and formulations and the ethical recommendations issued by the European Commission in 2008 and reviewed in 2017 [9].

In Europe, several collaborative initiatives have improved the knowledge and the quality of paediatric clinical research. They include the TEDDY European Network of Excellence for Paediatric Research, Global Research in Paediatrics Network (GRiP Network), several paediatric clinical research consortia funded under the Seventh Framework Programme, the European Network of Paediatric Research at the European Medicines Agency (Enpr-EMA) and all the specialty and national networks dedicated to paediatric clinical research.

In the field of clinical trials, a European Clinical Research Infrastructure Network exists (ECRIN-ERIC, www.ecrin.org) to provide tailored support to facilitate trial preparation and implementation, but it does not specifically address paediatric needs.

In 2016, the European Strategy Forum on Research Infrastructures recognised the need to setup a dedicated Paediatric Clinical Research Infrastructure and suggested to upgrade the existing research infrastructure (such as ECRIN-ERIC) to including paediatric topics [10].

PedCRIN is a four-year project funded by the European Union's Horizon 2020 programme, launched in 2017 to develop the necessary tools and capacity to support multinational, non-commercial paediatric clinical trials, based on the collaboration between the existing ECRIN-ERIC network and 14 relevant paediatric clinical research initiatives and networks in Europe.

PedCRIN project aims to develop the proposed tools according to the needs identified through a survey addressed to researchers involved in paediatric clinical research (paediatricians and neonatologist research communities).

The survey explicitly investigates the need of paediatric researchers to receive support from a research infrastructure and contributes PedCRIN project activities to focus on the mostly uncovered issues.

The aim of this paper is to report the results of this survey.

## 2 Materials and methods

### 2.1 Questionnaire design

A structured, cross-sectional, closed-ended questionnaire was developed. Yes-No questions, multiple choice questions and ordinal-scale questions were included. The questionnaire was drafted by a dedicated writing group, then circulated for comments and agreed within all the PedCRIN partnership (including ECRIN and the 14 European partners), and finalised.

To draft the questionnaire the following sources were considered: the CORBEL (Coordinated Research Infrastructures Building Enduring Life-science services) project [11]; the FP7 TINN (Treat Infection in Neonates) project questionnaire [12]; and the survey previously run by the TEDDY European Network of Excellence for Paediatric Research [13].

The final questionnaire consisted of three sections:

1. General section, asking for personal information, the country, the education profile, the therapeutic area of involvement.
2. Previous experience in paediatric clinical research (involvement and role in paediatric/neonatal clinical trials).
3. Needs for infrastructure services and tools for paediatric clinical trials, grouped into the following headings: 1.scientific and methodological expertise, 2. Clinical trials start-up, 3. Regulatory expertise, 4. Paediatric pharmacovigilance, 5. Conduct of paediatric clinical trials according to GCP and other paediatric guidelines/recommendations, and 6. Training.

Responders were asked to rate each item using a Likert-scale ranging from 0 (not needed) to 4 (extremely needed). For each heading, detailed subheadings were considered.

Final questionnaire is available in supplementary material.

### 2.2 Identification of the recipients

The survey was sent to members of the European paediatric healthcare and scientific community involved in paediatric/neonatal clinical trials and research: scientists participating in paediatric clinical research networks both at National and European level, representatives of Enpr-EMA networks (identified through the Enpr-EMA database) and other paediatric consortia (e.g., research consortia funded by the European Commission Research Framework Programmes and Horizon 2020, as available from the Cordis database), members of the coordinating/steering boards of scientific European societies dealing with paediatric research, representatives of European and national regulatory agencies and of the biomedical Research Infrastructures (identified through official websites).

### 2.3 Execution of the survey

The questionnaire was uploaded in a LimeQuery survey account (https://pedcrin.limequery.org) and identified recipients received by e-mail a link to complete the survey, together with an accompanying invitation message, specifying the purpose of the survey. A help-desk service was established (helpdesk@teddynetwork.net). The survey was open from April 4th up to May 15th, 2017. Periodic reminders for the completion were sent fortnightly.

### 2.4 Data analysis

Descriptive statistics were performed using SPSS version 21.0 (IBM Analytics) to describe all variables. Independent-Samples T-Test procedure comparing means was used to compare groups of answers (neonatologists and non-neonatologists, adherence to existing national networks or not). Analysis was repeated using Mann-Whitney *U* test, a non-parametric analogue of the two-samples *t*-test without the assumption of normally distributed data. Statistical significance was set at p < 0.05. Non-response bias was measured between early and late responses (assuming late respondent behave the same as non-respondents) based on outcome variables using the independent samples *t*-test (continuous variables) and the chi-square test (nominal variables).

## 3 Results

Our analysis allowed to identify 663 contacts. Following a data cleaning process, 65 invalid contacts (invalid e-mails, duplicates and general e-mail addresses not referring to a specific person) were removed, and 598 recipients from 46 countries received the electronic survey. Among them, 147 completed questionnaires were returned. The survey response rate was 24.6%. The *t*-test and the chi-square test for non-response bias analysis did not reveal significant difference between early (n = 54, 36.7%) and late respondents (n = 93, 63.3%) on any variable.

### 3.1 General information

**Fig. 1:**
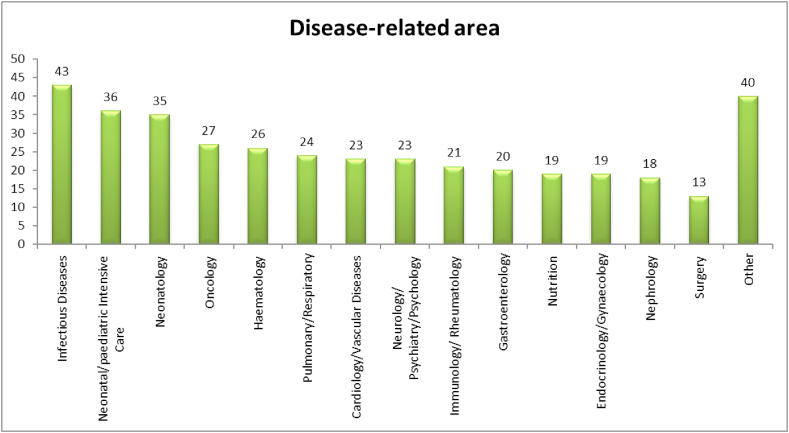
Disease-related area.

Respondents from 31 European and non-European countries answered the survey, with Spain and Italy being the most represented ones, followed by United Kingdom, the Netherlands and Germany. In terms of professional background, the qualification of paediatricians (57) and specialty paediatricians (58) were the most represented ones, followed by researchers (39) and Medical Doctors (36). Among respondents, there were also pharmacologists (13), pharmacists (15) and other experts (32) like research managers/coordinators, regulatory experts, research nurses. With reference to the specific therapeutic areas of expertise, the most common one was ‘infectious diseases’ (44/147, 29.9%). Neonatal expertise and paediatric/neonatal intensive care expertise were also well represented among respondents (35/147, 23.8% and 38/147, 25.8% respectively, Fig. 1).

‘Other’ includes: Paediatric formulations, Neurophysiology, pharmacology, toxicology, vaccination, anaesthesiology, Neglected Tropical Diseases, Epidemiology.

### 3.2 Clinical trials experience

Many respondents, 119 out of 147 (80.9%), declared being actively involved in paediatric clinical trials, with quite almost half of respondents (68/147, 46.2%) being involved in trials including pre-term and term neonates. Most of them acted as principal investigators/co-investigators, while 25 respondents were involved in clinical trials with other roles, namely scientific/medical coordinator, research nurse, pharmacist, study manager/supervisor, study assistant, medical writer, junior investigator, data manager, regulatory expert.

### 3.3 Rating of the need of support for paediatric clinical trials-related activities

For all the activities, most of respondents judged as high the need of support from a research infrastructure, i.e., very needed or extremely needed, for all the identified activities (Table 1).

**Table 1:** Respondents declaring the high/extreme need for support from a research infrastructure in paediatric clinical trials related activities, Independent-Samples *t*-test p-value.

Headings	Activities	Number (%) of Respondents declaring high need for support from a Research Infrastructure					QuestionnaireScore comparisonIndependent Samplest-test***p-value***
		Institutions adhering to National Networks (n = 91)		Institutions not adhering to National Networks (n=56)		Total (n = 147)	
		***N (%)***	**Questionnaire*Score(mean* ± *SD)***	***N (%)***	**Questionnaire*Score(mean*** ± ***SD)***	***N (%)***	
Scientific and methodological expertise	Design protocols for paediatric interventional clinical trials	70(76.9%)	3.02 ± 0.816	47 (83.9%)	3.09 ± 0.745	117(79.6%)	0.617
	Design protocols for paediatric non-interventional clinical studies	51(56%)	2.57 ± 0.909	33 (58.9%)	2.63 ± 0.945	84 (57.1%)	0.733
	Identification of the target population (age subsets, inclusion/exclusion criteria)*	40(44%)	2.31 ± 0.985	40 (71.4%)	2.75 ± 0.919	80 (54.4%)	0.008 *
	Statistical methodology for paediatric clinical trials*	60(65.9%)	2.76 ± 0.821	49 (87.5%)	3.29 ± 0.731	109 (74.1%)	<0.001 *
	Application of innovative study design (e.g. modeling& - simulation and extrapolation tools/approaches)	74 (81.3%)	3.14 ± 0.739	49 (87.5%)	3.36 ± 0.819	123 (83.7%)	0.104
Collaboration and support for clinical trial start-up	Identification of relevant network/scientific societies to help the selection of clinical trial sites	64 (70.3%)	2.85 ± 0.815	43 (76.8%)	3.02 ± 0.700	107 (72.8%)	0.193
	Establishing contacts with Young Persons Advisory Groups/Patients Advisory Boards/patients associations	55 (60.4%)	2.63 ± 0.915	36 (64.3%)	2.79 ± 0.825	91 (61.9%)	0.289
	Identification of relevant calls for funding paediatric trials at Eu/international level and support for application*	73 (80.2%)	3.00 ± 0.760	50 (89.3%)	3.29 ± 0.706	123 (83.7%)	0.025 *
	Involvement of parties and subcontractors to define the distribution of all the responsibilities/tasks in clinical trials	55 (60.4%)	2.60 ± 0.855	38 (67.9%)	2.84 ± 0.968	93 (63.3%)	0.126
	Preparation of standard model agreements for the implementation of clinical trials	63 (69.2%)	2.75 ± 0.902	42 (75%)	3.05 ± 0.980	105 (71.4%)	0.055
	Definition of a budget model based on standard costs for general activities, investigation, services, etc	64 (70.3%)	2.75 ± 0.938	41 (73.2%)	2.91 ± 0.859	105 (71.4%)	0.291
Regulatory expertise	Database of national regulatory and ethical requirements for paediatric trial authorization*	61 (67%)	2.73 ± 1.001	47 (83.9%)	3.11 ± 0.867	108 (73.5%)	0.020 *
	Preparing and submitting documents to Ethics Committess/Competent Authorities for the approval/authorization of paediatric clinical trials*	53 (58.2%)	2.60 ± 1.114	43 (76.8%)	3.02 ± 0.904	96 (65.3%)	0.015 *
	Preparing consent and assent models, information sheets*	57 (62.6%)	2.66 ± 1.046	45 (80.4%)	3.07 ± 0.951	102 (69.4%)	0.018 *
	Preparing the Investigator's Brochure for submission	52 (57.1%)	2.64 ± 0.949	40 (71.4%)	2.95 ± 0.883	92 (62.6%)	0.051
	Interaction with national/European regulatory agencies	67 (73.6%)	3.01 ± 0.850	44 (78.6%)	3.05 ± 0.749	111 (75.5%)	0.758
Paediatric pharmacovigilance	Methods for identifying and communicating Adverse Drug Reactions in paediatric patient*	55 (60.4%)	2.66 ± 1.046	44 (78.6%)	3.02 ± 0.726	99 (67.3%)	0.016*
	Age-adapted scales for severity and causality assessment in paediatric patients	62 (68.1%)	2.82 ± 0.961	40 (71.4%)	3.07 ± 0.912	102 (69.4%)	0.125
	Targeted Serious Adverse Events notification forms, age-adjusted*	59 (64.8%)	2.71 ± 1.047	45 (80.4%)	3.14 ± 0.773	104 (70.7%)	0.005 *
	Certification of pharmacovigilance expertise*	54 (59.3%)	2.67 ± 1.001	42 (75%)	3.04 ± 0.738	96 (65.3%)	0.012 *
Paediatric clinical trials conduct according to GCP and paediatric guidelines/recommendations	Design Case Report Forms for paediatric studies*	46 (50.5%)	2.45 ± 0.922	35 (62.5%)	2.79 ± 0.847	81 (55.1%)	0.029 *
	Managing paediatric clinical trial data (data-management)*	51 (56%)	2.57 ± 0.979	45 (80.4%)	3.00 ± 0.786	96 (65.3%)	0.004 *
	Managing paediatric Investigational Medicianal Products (drug management)	55 (60.4%)	2.65 ± 0.959	36 (64.3%)	2.86 ± 0.841	91 (61.9%)	0.182
	Managing paediatric clinical trial technical aspects & logistics	48 (52.7%)	2.52 ± 0.947	34 (60.7%)	2.79 ± 0.825	82 (55.8%)	0.081
	Preparation of monitoring plans, also based on risk-based approach	56 (61.5%)	2.70 ± 0.888	36 (64.3%)	2.80 ± 0.796	92 (62.6%)	0.491
	On-site and remote monitoring visits and reporting	48 (52.7%)	2.52 ± 0.993	33 (58.9%)	2.75 ± 0.815	81 (55.1%)	0.141
Training	GCP training, including responsibilities of principal investigators, co-investigators and study nurses involved in paediatric clinical trials*	50 (54.9%)	2.55 ± 0.946	40 (71.4%)	3.00 ± 0.991	90 (61.2%)	0.007 *
	Training course(s) designed for specific paediatric/neonatal trials*	58 (63.7%)	2.82 ± 0.889	45 (80.4%)	3.16 ± 0.781	103 (70.1%)	0.021*
	Training on drug safety and toxicity stratified by age*	56 (61.5%)	2.79 ± 0.863	44 (78.6%)	3.11 ± 0.779	100 (68.0%)	0.027*

More than 80% of respondents agreed on the relevance of having consultancy with a research infrastructure for the following items:

• Application of innovative studies design, like Modeling & Simulation or Extrapolation (123, 83.7%)
• Identification of relevant calls for funding paediatric trials at EU/international level and support for application (123, 83.7%)
• Design protocols for paediatric interventional clinical trials (117, 79.6%)

Full details are available in Table 1.

### 3.4 Neonatologists vs non-neonatologists

With reference to the opinion given by neonatologists, the trend of the answers was the same that of the other responders and no statistically significant differences emerged between the needs indicated by neonatologists and the ones that have been reported by the other respondents, not primarily focused on neonatology.

### 3.5 Centres adhering to a paediatric national network or not

The feedback from respondents from centres adhering to national networks (n = 91) was separately analysed. For 14 activities (marked with * in Table 1), needs reported from respondents not adhering to national networks were significantly higher than from the other group of respondents, especially in the fields of regulatory expertise, pharmacovigilance and Good Clinical Practice (GCP) training.

## 4 Discussion

For the purpose of the survey, the number of responses was considered satisfactory, and in line with the online response rate for an external survey (20–40%) [[14], [15], [16], [17]]. The survey was primarily addressed to people involved in ‘non-profit’ research. Thus, the results of the survey describe the needs of researchers involved in research-driven, not industry sponsored paediatric trials, that should be particularly interested in receiving support in term of planning services and tools for this specific setting. The sample of respondents is adequately representative and qualified to provide indications. The high rate of involvement in paediatric clinical trials, declared by the 81% of respondents, ensured that their contribution reflect the needs of a community strongly and actively engaged in survey topics. These considerations are consistent with results of non-response bias analysis.

### 4.1 Areas of major needs

All the needs categories mentioned in the survey have been strongly acknowledged and perceived with no clear prevalence of an item on the other ones.

These results confirmed the original expectation that clinical paediatric trials require careful evaluation and qualified support all-over the process. However, the survey results indicate some specific areas of interest that would represent a guide to prioritize the perceived gaps.

Firstly, it was noted that the preparation of protocols for paediatric intervention clinical trials, including the application of innovative approaches in the design of studies, got the highest frequency of high need for support. Remarkably, for the application of innovative study design, 40% of respondents reported the need for support as ‘extreme’.

This high percentage could be largely attributed to the prevalence of respondents belonging to the scientific sector (academy, research centres, scientific societies), who usually lack of adequately structured training on drug development issues and clinical research methodology during the academic courses.

This also confirms the validity of the ECRIN model mainly devoted to support academic research activities as well as the need of similar initiatives to be conducted in the paediatric sector.

The identification and application to calls for funding has also been acknowledged as a relevant gap for researchers, since it requires a series of competences (budget, management, communication/dissemination, etc) that are not straightforward part of the scientific curriculum [18].

Another relevant gap is represented by the difficulties mentioned by our respondents in establishing and maintaining collaboration with regulators and Ethics Committees that might be linked to the difficulties from Academy and Research Centres/Hospitals to be compliant with the regulatory and ethical requirements and the poor availability and flexibility from Ethics Committees/regulators for clarification and support.

To reduce this gap, the PedCRIN contribution could be relevant. In fact, PedCRIN dedicated a project Work Package (WP3) to implement existing ECRIN instruments by introducing paediatric adaptations and to develop new tools for paediatric trials. Among these:

- the CAMPUS Database – launched in December 2015 by ECRIN and currently in updating process – is an online database including country-specific information on regulatory and ethical requirements in clinical research across Europe. The CAMPUS Database currently includes information on clinical research in medicinal products for human use, medical devices, and/or nutrition for over 22 European countries. In the framework of PedCRIN, information on clinical studies in the paediatric and neonatal population have been added with the contribution of PedCRIN partners.; Other relevant tools have been proposed or updated starting from existing products already designed in the framework of other paediatric initiatives. They cover with paediatric specifity the following items: biosample management, study design (including designs for small sample size), sample size estimation, choice of comparator, harmonized terminology, outcomes selection, measurement and report, trial monitoring plan, pharmacovigilance, trial conduct according to GCP and guidelines; specific tools have been foreseen for neonatal trials, including training aspects, ethics and pharmacokinetics specificities, design methodology and outcome measurement in the setup of neonatal trials.

Concerning training, it should be underlined that high-level educational courses in the field of regulatory science at academic level are lacking. The experience conducted within the Global Research in Paediatric (GRiP) project [19] demonstrated that an ad hoc high-level course tailored on paediatric clinical trials is feasible, while complex. In addition, thanks to the projects funded by the European Commission to develop research-driven trials as part of a regulatory process (Seventh Framework Programme, Health 2007–2013, topic 4.2–1 “to develop off-patent medicinal products for the paediatric population”), it has been also demonstrated the capabilities of the paediatric community involved in the projects to committee itself into methodological and regulatory procedures with positive results. These contributions are encouraging and support the need to set up academic master courses or other high level training initiatives, encountering the specific need of paediatrics and neonatologist involved in clinical research, using dedicated funding and stimulating the scientific collaboration between industry and academy [20,21].

Another relevant result of our study is the demonstrated progressive reduction of the regulatory and methodological gaps expressed by researchers that operate in the context of a paediatric clinical research National Networks, respect to the other centres. This is particularly true for some activities, including regulatory procedures, pharmacovigilance and GCP-training. National paediatric trials networks (like the Spanish Pediatric Clinical Trials Network RECLIP, the Italian Network for Pediatric Clinical Trials INCiPiT, the Finnish Investigators Network for Pediatric Medicines FinPedMed, the National Institute for Health Research Medicines for Children Research Network in UK, etc.) have been recently set up in EU to support the initiatives of researchers and academy and to participate actively to c4c, the IMI2 funded collaborative network for European clinical trials for children [22]. In this context, the National Networks are developing qualified support services and are providing reliable source of information for paediatric clinical research in their reference countries.

In conclusion, this survey experience confirms that methodological, funding, training and regulatory gaps are still documented in the field of paediatric clinical research. Initiatives as PedCRIN, in conjunction with other ongoing paediatric initiatives, are offering valid instrument to increase performances and capacities of clinical centres in running paediatric clinical research. Considering that clinical trial performance requires centres to evolve their organisation also beyond the clinical aspects, any structured support that might be given from a research infrastructure is expected to be welcome.

### 4.2 Strengths and limitations of this study

The geographical distribution has been acknowledged as the main limitation of this study, with 51.7% of answers from five countries (Spain, Italy, United Kingdom, Germany and the Netherlands). This mostly depended on the survey promotion among the national networks (e.g. the Italian INCiPiT and the Spanish RECLIP, accounting for 28.5% of answers).

Response rate can be higher with more time to run the survey, however, this experience describes the needs of almost 150 experts in paediatric clinical research across Europe, working in different fields and institutions.

The qualified profile of respondents, as highlighted by their direct involvement in paediatric clinical trials, also including the neonatal population, increases the reliability of our data.

This study represents the first wide-ranging experience to investigate the needs of the experts involved in paediatric clinical research.

## 5 Conclusion

In conclusion, the results of this survey demonstrated the need of structured support to paediatric research. PedCRIN activities and tools would get a promising feedback from users, provided that the experience will continue after the end of the project, in collaboration with the other existing qualified paediatric research initiatives and the changed research framework. To reach this goal, cooperation with biomedical research infrastructures, clinical trials specialty networks, national hubs and large collaborative research initiatives should be supported. This will finally allow to: integrate the paediatric specificities in all the stages of research; connect the most relevant centres and experts among Europe to improve collaborative research; push for support and structured funding from national and EU bodies; covering the whole pathway of paediatric research.

## Authors’ contributions

All the authors were involved in critically revising the manuscript for important intellectual content and they have all approved this final version. LR (study design and conduct, questionnaire preparation, manuscript drafting). AC, DB, SM, VE & MF (collaboration in questionnaire preparation, contribution to study design & manuscript review), DB, JD, EJA (collaboration in study design, manuscript review), FB (technical collaboration in study conduct), ML & CM (collaboration in questionnaire preparation) & GR (statistical analysis).

## Funding

The PedCRIN project has received funding from the European Union's Horizon 2020 research and innovation programme, under grant agreement n° 731046.

## Compliance with ethical standards

The survey did not study any individual patient data. Therefore, no ethical approval was requested, and informed consent was not applicable.

## Declaration of competing interest

The authors declare that they have no competing interests.
