# Supplementary material for: Paediatric clinical research in Europe: an insight on experts’ needs and perspectives
Source: Contemp Clin Trials Commun. 2021 Feb 10;21:100735. doi: 10.1016/j.conctc.2021.100735 (PMC7905444; doi:10.1016/j.conctc.2021.100735)
Supplement: Multimedia component 1 [file mmc1.docx]

# Supplementary material – Survey form

**PedCRIN - Survey on infrastructure and service needs for paediatric and neonatal trials**

Dear Colleague,

The H2020-funded project PedCRIN (Paediatric Clinical Research Infrastructure Network, G.A. 731046) has recently launched its activities to enrich the ECRIN tools and actions with paediatric specificities.
In order to optimize the forthcoming PedCRIN services and activities, we sought your collaboration in the identification of the needs and expectations of paediatric medical research communities.
For this purpose, we kindly ask you to complete the following brief questionnaire (5 minutes).
Please do not hesitate to contact us at the following e-mail address ([helpdesk@teddynetwork.net](mailto:helpdesk@teddynetwork.net)) for any question about the survey and any further information.

Thank you in advance for your collaboration.

Donato Bonifazi

PedCRIN WP3 Leader

**I. GENERAL**

- **Your name ***
- **Your organisation ***
- **Your country ***
- **Email address ***
- **Your profile ***

Please choose **all** that apply:

- Paediatrician
- Specialty paediatrician (please specify the specialty):
- Medical Doctor
- Researcher from academy/research centre
- Pharmacologist
- Pharmacist
- Other (please specify):
- **Your disease-related area ***

Please choose **all** that apply:

- Neonatology
- Neonatal/paediatric Intensive Care
- Cardiology/Vascular Diseases
- Endocrinology/Gynaecology
- Gastroenterology
- Haematology
- Immunology/ Rheumatology
- Infectious Diseases
- Nephrology
- Neurology/ Psychiatry/Psychology
- Nutrition
- Oncology
- Pulmonary/Respiratory
- Surgery
- Other:

**II. PREVIOUS EXPERIENCE IN PAEDIATRIC CLINICAL RESEARCH**

- **Did you actively take part in any clinical trials involving paediatric subjects (0-18 years)? ***

🞎 Yes 🞎 No

- **Did any of the trials you took part in involve pre-term and term neonates? ***

**Only answer this question if** answer was 'Yes' at question 'Did you actively take part in any clinical trials involving paediatric subjects (0-18 years)?’

🞎 Yes 🞎 No

- **Please specify your role in the clinical trial ***

**Only answer this question if** answer was 'Yes' at question 'Did you actively take part in any clinical trials involving paediatric subjects (0-18 years)?’

Please choose **all** that apply:

- Principal Investigator
- Co-investigator
- Other:

**III. NEEDS FOR INFRASTRUCTURE SERVICES AND TOOLS FOR PAEDIATRIC CLINICAL TRIALS**

Research infrastructures (RIs) provide services to the scientific community to conduct top-level research in their respective field. PedCRIN is supported by the European Commission to develop services and tools for paediatric trials within ECRIN ([www.ecrin.org](http://www.ecrin.org)). This questionnaire will be used to establish priorities in the development of paediatric clinical research tools by PedCRIN.

- **Please indicate, for which of the following activities do you think a research infrastructure for paediatric clinical research should provide support to?**

**SCIENTIFIC AND METHODOLOGICAL EXPERTISE**

Please choose the appropriate response for each item:

|  | 0  No need at all | 1  Slightly needed | 2  Moderately needed | 3  Very needed | 4  Extremely needed |
| --- | --- | --- | --- | --- | --- |
| Design protocols for paediatric interventional clinical trials (PK, PK/PD, efficacy and/or safety, other) | 🞎 | 🞎 | 🞎 | 🞎 | 🞎 |
| Design protocols for paediatric non-interventional clinical studies | 🞎 | 🞎 | 🞎 | 🞎 | 🞎 |
| Identification of the target population (age subsets, inclusion/exclusion criteria) | 🞎 | 🞎 | 🞎 | 🞎 | 🞎 |
| Statistical methodology for paediatric clinical trials | 🞎 | 🞎 | 🞎 | 🞎 | 🞎 |
| Application of innovative study design (e.g. modeling&simulation and extrapolation tools/approaches) from adults to children and from older children to neonates | 🞎 | 🞎 | 🞎 | 🞎 | 🞎 |

**COLLABORATION AND SUPPORT FOR CLINICAL TRIALS START-UP**

Please choose the appropriate response for each item:

|  | 0  No need at all | 1  Slightly needed | 2  Moderately needed | 3  Very needed | 4  Extremely needed |
| --- | --- | --- | --- | --- | --- |
| Identification of relevant network/scientific societies to help the selection of clinical trial sites | 🞎 | 🞎 | 🞎 | 🞎 | 🞎 |
| Establishing contacts with Young Patients Advisory Groups/Patients Advisory Boards/Patients Associations | 🞎 | 🞎 | 🞎 | 🞎 | 🞎 |
| Identification of relevant calls for funding paediatric trials at Eu/international level and support for project application | 🞎 | 🞎 | 🞎 | 🞎 | 🞎 |
| Involvement of parties and subcontractors to define the distribution of all the responsibilities and tasks related to clinical trials (including CROs, insurance companies, etc) | 🞎 | 🞎 | 🞎 | 🞎 | 🞎 |
| Preparation of standard models agreements for the implementation of clinical trials | 🞎 | 🞎 | 🞎 | 🞎 | 🞎 |
| Definition of a budget model based on standard costs for general activities, investigation (per patient), services, etc | 🞎 | 🞎 | 🞎 | 🞎 | 🞎 |

**REGULATORY EXPERTISE**

Please choose the appropriate response for each item:

|  | 0  No need at all | 1  Slightly needed | 2  Moderately needed | 3  Very needed | 4  Extremely needed |
| --- | --- | --- | --- | --- | --- |
| Database of national regulatory and ethical requirements for paediatric trial authorisation | 🞎 | 🞎 | 🞎 | 🞎 | 🞎 |
| Preparing and submitting documents to Ethics Committees/Competent Authorities for the approval/authorisation of paediatric clinical trials | 🞎 | 🞎 | 🞎 | 🞎 | 🞎 |
| Preparing consent and assent models + Patient information sheet, including clinical trials involving special patients populations (PICU, NICU, neonates, neurological impairment, etc) | 🞎 | 🞎 | 🞎 | 🞎 | 🞎 |
| Preparing the Investigator’s Brochure for submission | 🞎 | 🞎 | 🞎 | 🞎 | 🞎 |
| Interaction with national/European regulatory agencies | 🞎 | 🞎 | 🞎 | 🞎 | 🞎 |

**PAEDIATRIC PHARMACOVIGILANCE**

Please choose the appropriate response for each item:

|  | 0  No need at all | 1  Slightly needed | 2  Moderately needed | 3  Very needed | 4  Extremely needed |
| --- | --- | --- | --- | --- | --- |
| Methods for identifying and communicating ADRs in paediatric patients | 🞎 | 🞎 | 🞎 | 🞎 | 🞎 |
| Age-adapted scales for severity and causality assessment in paediatric patients | 🞎 | 🞎 | 🞎 | 🞎 | 🞎 |
| Targeted Serious Adverse Events notification forms, age-adjusted | 🞎 | 🞎 | 🞎 | 🞎 | 🞎 |
| Certification of pharmacovigilance expertise | 🞎 | 🞎 | 🞎 | 🞎 | 🞎 |

**PAEDIATRIC CLINICAL TRIALS CONDUCT ACCORDING TO GCP AND PAEDIATRIC GUIDELINES/RECOMMENDATIONS**

Please choose the appropriate response for each item:

|  | 0  No need at all | 1  Slightly needed | 2  Moderately needed | 3  Very needed | 4  Extremely needed |
| --- | --- | --- | --- | --- | --- |
| Design Case Report Forms for paediatric studies | 🞎 | 🞎 | 🞎 | 🞎 | 🞎 |
| Managing paediatric clinical trial data (data-management) (collection, integration, validation and analysis of clinical trial data) | 🞎 | 🞎 | 🞎 | 🞎 | 🞎 |
| Managing paediatric IMPs (drug management) (packaging, labelling, delivering, storing, administering, accountability, disposal) | 🞎 | 🞎 | 🞎 | 🞎 | 🞎 |
| Managing paediatric clinical trial technical aspects & logistics (e.g. shipping agent, operative instructions, laboratory procedures, biobank samples management, etc.) | 🞎 | 🞎 | 🞎 | 🞎 | 🞎 |
| Preparation of monitoring plans, also based on risk-based approach | 🞎 | 🞎 | 🞎 | 🞎 | 🞎 |
| On-site and remote monitoring visits and reporting | 🞎 | 🞎 | 🞎 | 🞎 | 🞎 |

**TRAINING**

Please choose the appropriate response for each item:

|  | 0  No need at all | 1  Slightly needed | 2  Moderately needed | 3  Very needed | 4  Extremely needed |
| --- | --- | --- | --- | --- | --- |
| Training regarding Good Clinical Practices, including responsibilities of principal investigators, co-investigators and study nurses involved in paediatric clinical trials | 🞎 | 🞎 | 🞎 | 🞎 | 🞎 |
| Training course(s) designed for specific paediatric/neonatal trials | 🞎 | 🞎 | 🞎 | 🞎 | 🞎 |
| Training on drug safety and toxicity stratified by age | 🞎 | 🞎 | 🞎 | 🞎 | 🞎 |

- **Please list any other activity for which do you think that it is required support from a research infrastructure:**
